# Non-convulsive Status Epilepticus in L-2-Hydroxyglutaric Aciduria With a Novel L2HGDH Variant: A Case Report

**DOI:** 10.7759/cureus.114093

**Published:** 2026-08-06

**Authors:** Sarthak Mittal, Srinath Rajeevan, Siby Gopinath, Akshaya Raman

**Affiliations:** 1 Neurology, Amrita Institute of Medical Sciences, Kochi, IND; 2 Neurology, Amala Institute of Medical Sciences, Thrissur, IND; 3 Neurotechnology/Neurology, Amrita Institute of Medical Sciences, Kochi, IND

**Keywords:** ataxia, epileptiform discharges, l2hgdh mutation, missense variant, non-convulsive status epilepticus, rare neurometabolic disorder

## Abstract

L-2-hydroxyglutaric aciduria (L2HGA) is a rare autosomal recessive neurometabolic disorder caused by variants in the L2HGDH gene, typically presenting with developmental delay, cognitive impairment, seizures, movement disorders, and progressive ataxia. We report the case of a 29-year-old man with childhood-onset epilepsy and neurodevelopmental impairment who presented with recurrent episodes of behavioral arrest and unresponsiveness. Video electroencephalography demonstrated frequent right temporal electrographic seizures that fulfilled the Salzburg criteria for non-convulsive status epilepticus (NCSE). Brain MRI showed a characteristic pattern of predominantly subcortical and deep white matter abnormalities with bilateral dentate nucleus and basal ganglia involvement, supporting a diagnosis of L2HGA.

Biochemical evaluation was atypical, with elevated ethylmalonic and methylsuccinic acids and abnormalities in the acylcarnitine profile. Long-term valproate exposure was considered a potential contributor to these findings. Quantitative L-2-hydroxyglutarate estimation was unavailable. Whole-exome sequencing identified a previously unreported homozygous L2HGDH missense variant, c.191C>A (p.Ala64Asp), currently classified as a variant of uncertain significance (VUS). The variant was absent from the population databases queried, while multiple in silico tools, including REVEL (0.96), AlphaMissense (0.994), and SIFT (0.001), predicted a deleterious effect. This case broadens the recognized seizure phenotype of L2HGA by highlighting NCSE as a potential presentation and illustrates the complementary role of molecular testing when biochemical evaluation is atypical or incomplete.

## Introduction

L-2-hydroxyglutaric aciduria (L2HGA) is a rare autosomal recessive neurometabolic disorder. It is caused by variations in the L-2-hydroxyglutarate dehydrogenase gene (L2HGDH, OMIM: 609584), located on chromosome 14. The L2HGDH enzyme is a flavin adenine dinucleotide (FAD)-linked mitochondrial enzyme that catalyzes the irreversible conversion of 2-hydroxyglutarate (L2HG) to alpha-ketoglutarate [1-3]. Defective L2HGDH enzyme leads to accumulation of 2-hydroxyglutarate, which causes neurotoxicity via oxidative stress. In addition, L2HGA is increasingly recognized as a metabolite repair disorder in which the accumulation of L2HG leads to mitochondrial dysfunction and excitotoxic neuronal injury mediated by NMDA receptor overstimulation [1-5].

Patients typically present with developmental delay, cognitive impairment, seizures, movement and gait disorders, and behavioral disturbances. The disease usually has insidious onset and progression, while acute metabolic decompensation or rapid neurological deterioration is uncommon [6,7]. Clinical manifestations typically begin in childhood and follow a slowly progressive course, although the variable phenotype and indolent progression may result in diagnosis during adolescence or adulthood [7,8]. Diagnosis is confirmed at three levels: imaging, biochemical, and genetic. MRI typically shows a centripetal pattern of white matter hyperintensities, dentate nucleus, and basal ganglia with cerebellar volume loss. Periventricular, commissural, brainstem, and thalamic structures are spared [9-11]. Biochemically, elevated L-2-hydroxyglutaric acid in the serum and urine is diagnostic [12], followed by genetic confirmation with the identification of L2HGDH gene mutation [13].

More than 70 pathogenic genetic variants in the L2HGDH gene have been described, with significant allelic heterogeneity and clustering of variants in certain populations, without a single globally dominant variant [13]. In patients with neurometabolic disorders, non-convulsive status epilepticus (NCSE) may be difficult to recognize clinically because behavioral arrest and fluctuating encephalopathy may be attributed to the underlying metabolic abnormality, making electroencephalography (EEG) essential. Although seizures are recognized in L2HGA, NCSE remains poorly characterized and may be under-recognized. We report a novel case of L2HGA associated with NCSE and a novel L2HGDH variant.

## Case presentation

A 29-year-old male, born to non-consanguineous parents by term caesarean section, presented with multiple daily episodes of transient unresponsiveness and behavioral arrest for the preceding two weeks. During these events, lasting 30 seconds to one minute, he was unresponsive to commands, without associated tonic-clonic activity, myoclonus, drop attacks, tongue biting, or incontinence, and recovered his baseline sensorium between episodes. His history revealed episodic seizures from six months of age. He had received anti-seizure medication since childhood; however, details of his earlier medication, doses, and duration were unavailable.

**Figure 1:**
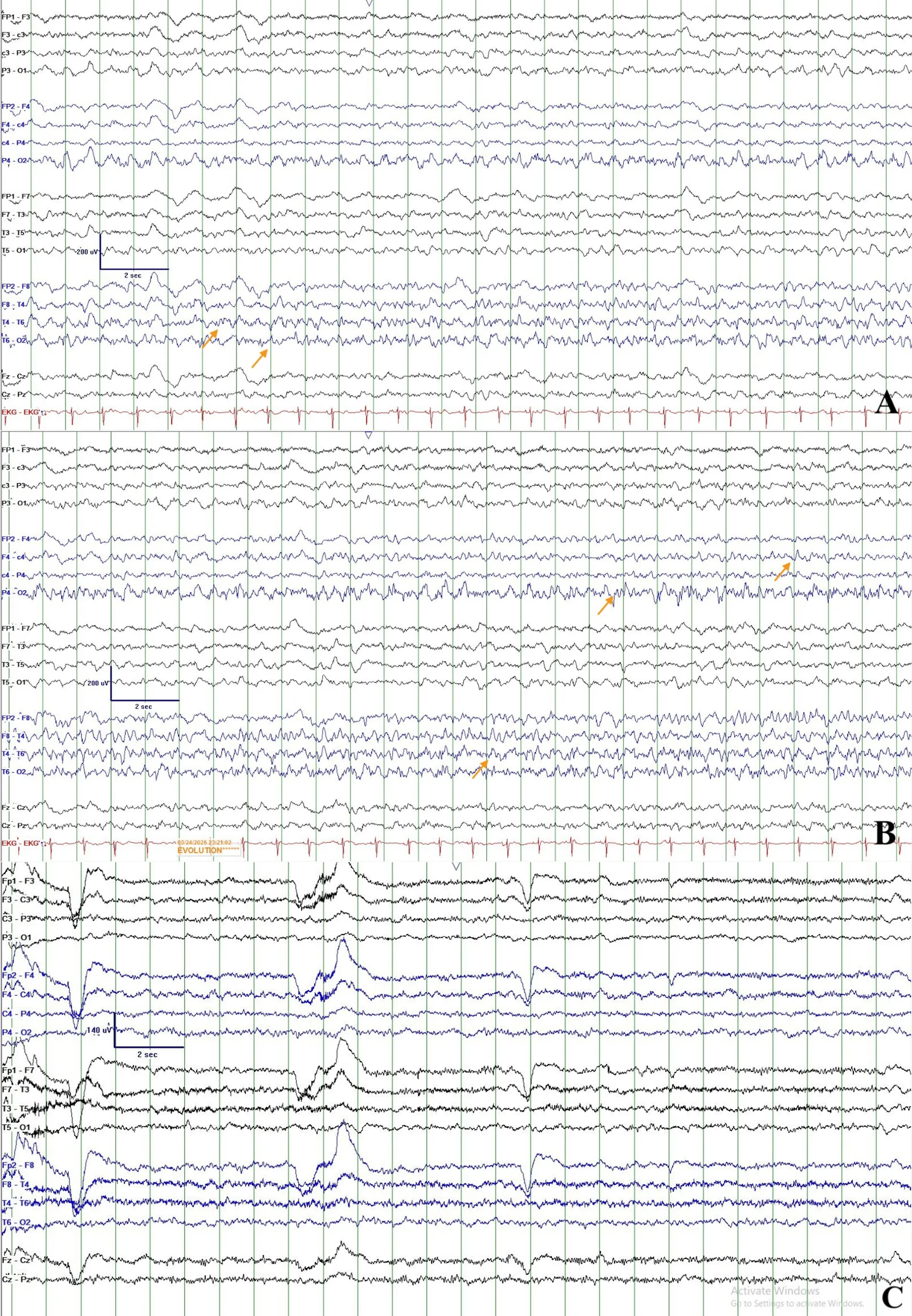
EEG findings (A) Bipolar montage showing onset of seizures in the form of rhythmic activity from the right middle and posterior temporal leads as indicated by the solid golden arrow. Paper speed: 30 mm/sec; sensitivity: 7 µv; HFF: 70 Hz; LFF: 0.5 Hz; Notch: 50 Hz. (B) Bipolar montage showing propagation of seizures to the right occipital and right fronto-central leads as indicated by the solid golden arrow. Paper speed: 30 mm/sec; sensitivity: 7 µv; HFF: 70 Hz; LFF: 0.5 Hz; Notch: 50 Hz. (C) Bipolar montage showing termination of seizures after administration of midazolam. Paper speed: 30 mm/sec; sensitivity: 7µv; HFF: 70 Hz; LFF: 0.5 Hz; Notch: 50 Hz EEG: electroencephalography; HFF: high-frequency filter; LFF: low-frequency filter

At presentation, he was receiving valproate (500 mg twice daily), which had reportedly provided prolonged seizure control. His birth history was unremarkable; however, developmental delay in speech and poor scholastic performance were noted. He had remained seizure-free between the ages of 8 and 23 years, after which he developed generalized tonic-clonic (GTCS) seizures (two to three annually), again without a detailed evaluation. Examination showed cerebellar impairment, spasticity, dystonia, choreiform upper-limb movements, and hypometric saccades. His gait was broad-based, requiring support due to worsening ataxia. Clinically, an ataxia-dystonia syndrome with hyperkinetic movements was diagnosed. The transient episodes of impaired awareness suggested focal seizures with impaired awareness. Video EEG (vEEG) recorded multiple electrographic seizures (Figure 1), which responded to midazolam, consistent with NCSE as per the Salzburg criteria [10]. Seizure control required levetiracetam, clobazam, and lacosamide.

**Figure 2:**
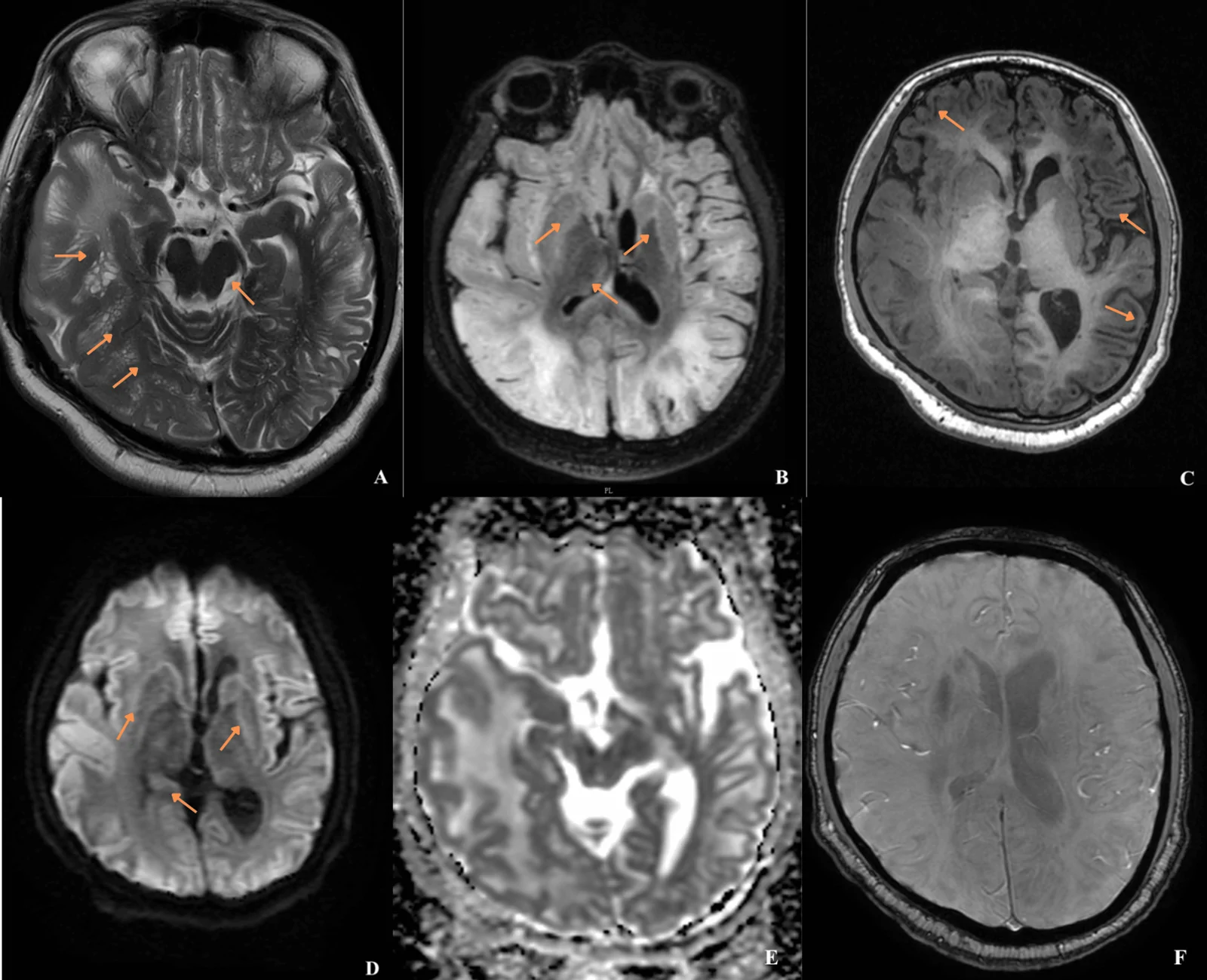
MRI findings (A) T2-weighted images show diffuse involvement of the deep and subcortical white matter along with cystic changes and prominent perivascular spaces. The brainstem is characteristically spared. (B) FLAIR imaging shows the characteristic centripetal pattern of involvement with involvement of the deep and subcortical white matter and bilateral basal ganglia and right thalamus. Periventricular white matter and corpus callosum are spared. (C) T1-weighted image shows diffusely hypointense white matter with U-fibre involvement. Periventricular white matter is spared. (D) DWI is consistent with FLAIR imaging, with no focus of diffusion restriction (E) or blooming (F) MRI: magnetic resonance imaging; FLAIR: fluid-attenuated inversion recovery; DWI: diffusion-weighted imaging

At the time of presentation, vital parameters, including blood pressure (BP: 110/70 mmHg), pulse rate (PR: 90 per minute), respiratory rate (RR: 16 per minute), and blood glucose measured by glucometer (97 mg/dL), were normal. The patient was of a lean build; however, detailed anthropometric data were unavailable. Routine blood investigations, including serum ammonia, were normal, as shown in Table 1. CSF analysis revealed a normal opening pressure, two mononuclear cells, normal sugar, and slightly elevated protein (68.2 mg/dL). The CSF protein elevation was nonspecific and, in the absence of pleocytosis or other inflammatory findings, could not be attributed specifically to L2HGA or NCSE. MRI of the brain revealed diffuse T2/FLAIR hyperintensities predominantly involving the deep and subcortical white matter, with bilateral dentate nucleus, basal ganglia, and right thalamic involvement. Sparing of the periventricular white matter, corpus callosum, and internal capsule, with preserved cerebellar volume, produced the characteristic pattern of L2HGA (Figure 2).

**Table 1:** Lab parameters at the time of admission CRP: C-reactive protein; TLC: total leukocyte count; PT: prothrombin time; INR: international normalized ratio; AST: aspartate aminotransferase; ALT: alanine transaminase; ALP: alkaline phosphatase

Parameters	Value
CRP	0.43 mg/dL
Hemoglobin	15.3 g/dL
Platelet count	2,22,000/uL
TLC	7,990/uL
PT/INR	14.7 seconds/1
AST	14 IU/L
ALT	21.9 IU/L
ALP	50 IU/L
Total bilirubin	0.61 mg/dL
Urea	36.6 mg/dL
Creatinine	1.01 mg/dL
Sodium	136.6 mmol/L
Potassium	3.7 mmol/L
Magnesium	2.3 mg/dL
Phosphorous	3.3 mg/dL
Calcium	9.38 mg/dL
Ammonia	35.3 umol/L
Ceruloplasmin	18.65 mg/dL

Quantitative estimation of L-2-hydroxyglutaric acid could not be performed. Gas chromatography-mass spectrometry (GCMS) revealed elevated ethylmalonic and methylsuccinic acids, along with valproate metabolites, indicating an atypical biochemical profile. The acylcarnitine profile demonstrated elevated C4 levels (3.17 µmol/L; reference: < 1.30), along with increased C4/C2 (0.24; reference: < 0.10) and C4/C3 ratios (1.17; reference: < 0.70), providing evidence of altered intermediary metabolism. Despite the atypical biochemistry, whole exome sequencing identified a homozygous missense variant of uncertain significance (VUS), c.191C>A (p.Ala64Asp), in exon 2 of the L2HGDH gene (chr14:g.50302967G>T), absent from the 1000 Genomes, gnomAD, TopMed, and MedGenome databases. In silico prediction tools demonstrated a consistent deleterious effect, with a REVEL score of 0.96 and an AlphaMissense score of 0.994. SIFT predicted the variant to be damaging (score 0.001), and additional tools (DANN, MetaLR, and BayesDel) supported a deleterious effect.

**Figure 3:**
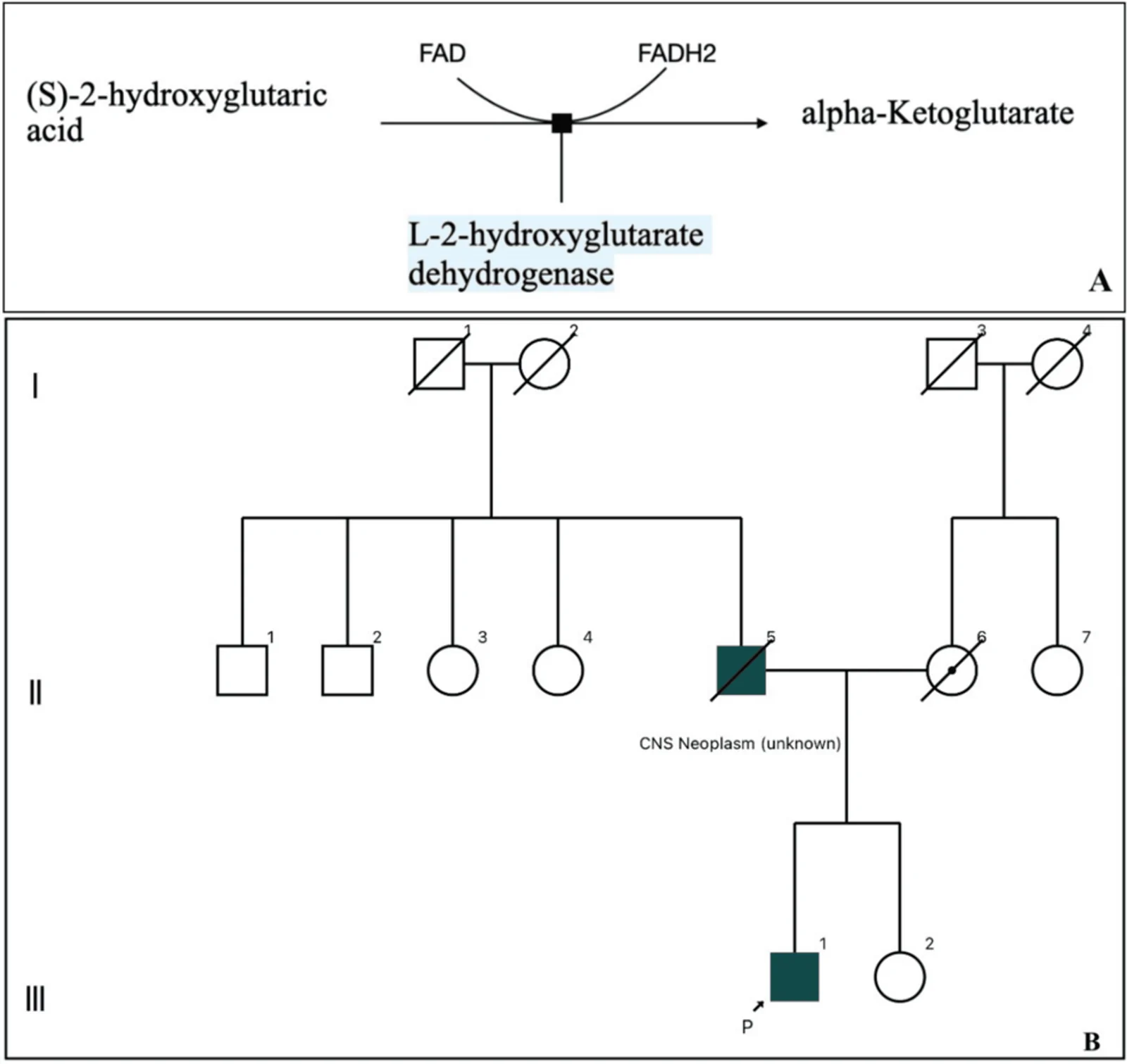
Metabolism and pedigree chart (A) FAD-dependent metabolism of (S)-2-hydroxyglutarate to alpha-ketoglutarate by the L2HGDH enzyme [12]. (B) Pedigree chart - our patient is the proband. The chart depicts a cerebral neoplasm of unknown etiology in the father of our patient. Details about the uncles and aunts were not available. Whole exome sequencing was also done for the sister, which was negative FAD: flavin adenine dinucleotide; L2HGDH: L-2-hydroxyglutarate dehydrogenase

Based on the American College of Medical Genetics and Genomics (ACMG) criteria, the variant was classified as a variant of uncertain significance (PM2: absent from population databases; PP3: multiple computational predictions; PP2: missense variant in a gene with a low benign variation rate) [14]. Mitochondrial genome sequencing was normal. Whole exome sequencing of the patient's sister was negative for the same mutation. No definitive familial association could be established (Figure 3B).

The patient received riboflavin (100 mg/day), levocarnitine (1 g/day), and folic acid (10 mg/day). At the one-month follow-up, he was more alert, and his family reported a reduction in seizure frequency. Follow-up EEG continued to show interictal epileptiform discharges. Because the antiseizure therapy had also been modified, and no standardized neurological or biochemical outcome measures were obtained, the improvement could not be attributed specifically to riboflavin, levocarnitine, or folic acid.

## Discussion

L2HGA is a cerebral organic acid disorder characterized as a metabolite repair defect leading to the accumulation of neurotoxic metabolites. Hallmark features of macrocephaly, intellectual disability, cerebellar and extrapyramidal signs, and distinctive MRI findings should prompt evaluation [6,15]. The MRI of our patient exhibited the classical centripetal pattern of white matter involvement, with sparing of the periventricular, commissural, and brainstem regions, as previously described in large cohorts [9-11]. Cystic degeneration and volume loss may accompany these changes, while the dentate nuclei and basal ganglia are typically involved. Such imaging prompted genetic testing despite the atypical biochemistry [7,16].

Acute and recurrent encephalopathy is occasionally observed in L2HGA, often triggered by seizures, status epilepticus, or metabolic stressors [8,10,17]. To our knowledge, NCSE has not previously been specifically reported as a manifestation of L2HGA. Our patient’s EEG demonstrated rhythmic fast activity evolving to rhythmic theta over the right temporal region (Figure 1), fulfilling the Salzburg Consensus Criteria for NCSE [18,19]. One possible explanation is that cortico-subcortical network dysfunction due to white matter and basal ganglia involvement may predispose patients to non-convulsive seizure states. This expands the known seizure spectrum of L2HGA, highlighting the importance of EEG in patients with fluctuating encephalopathy.

Although elevated L-2-hydroxyglutaric acid is the biochemical hallmark [12], our patient demonstrated ethylmalonic acid (EMA) elevation on GCMS, which is atypical for L2HGA. He was receiving long-term valproate, a mitochondrial β-oxidation inhibitor known to elevate EMA and unmask latent mitochondrial disorders. Valproate is known to inhibit mitochondrial β-oxidation, leading to the accumulation of toxic intermediates and secondary metabolic derangements. Valproate can impair mitochondrial β-oxidation and carnitine-dependent fatty acid metabolism, resulting in secondary organic acid and acylcarnitine abnormalities. In a patient with an underlying metabolite repair disorder, such mitochondrial metabolic stress could plausibly accentuate metabolic vulnerability. However, a direct interaction between valproate exposure and L2HGDH deficiency has not been established [6,16,17].

Multiple L2HGDH mutations have been catalogued in the Leiden Open Source Variation Database and ClinVar [20,21]. Although the c.191C>A (p.Ala64Asp) variant remains classified as a VUS according to ACMG criteria, several findings support its potential clinical relevance: its homozygous state in a patient with a characteristic L2HGA phenotype and neuroimaging pattern, its absence from the population databases queried, and concordant deleterious predictions across multiple in silico tools. Nevertheless, in the absence of functional studies, segregation evidence sufficient for reclassification, or previous reports of the variant in affected individuals, its pathogenicity cannot be established from the present case.

Unique aspects of this case include the presence of NCSE, a novel L2HGDH variant, and an atypical metabolic profile with elevated EMA. The described picture may have developed as a consequence of the interplay between underlying mitochondrial vulnerability, valproate toxicity, and mitochondrial dysfunction. This case highlights the complementary role of molecular testing in metabolite repair disorders, particularly when the biochemical profile is atypical or incomplete.

## Conclusions

This case report describes NCSE in an adult with a characteristic clinical and neuroimaging phenotype of L2HGA and a previously unreported homozygous L2HGDH variant (c.191C>A; p.Ala64Asp), currently classified as a VUS. The atypical biochemical profile, including elevated EMA and acylcarnitine abnormalities, may have been influenced by long-term valproate exposure. The report broadens the recognized seizure phenotype of L2HGA and highlights the importance of EEG evaluation in patients with fluctuating encephalopathy, as well as the complementary role of molecular testing when biochemical evaluation is atypical or incomplete.
